# Comparison of Clinical Effectiveness for Lumbar Pain Reduction between Exercise Programs on Flat Surface and Proprioceptive Equipment

**Published:** 2019-11

**Authors:** Woo Yeul BAEK, Sang Soo KIM, Sung Bum JU

**Affiliations:** 1. Department of Sport Management, Kyonggi University, Suwon, South Korea; 2. Department of Physical Education, Keimyung University, Daugu, South Korea; 3. Department of Physical Education, Busan National University of Education, Busan, South Korea

## Dear Editor-in-Chief

Low back pain (LBP) is the persistent pain of lumbar muscle and vertebra more than three days regardless of the existence of radiating pain to lower limbs (1). The symptom of LBP is caused by the hypokinesia of muscle and tendon supporting the lumbar vertebra and, in turn, lumbar muscle dystrophy caused by the inactivity of the lumbar vertebra leads to aggravated lumbar pain and pain recurrence (2).

In order to treat LBP, an exercise therapy is more effective for lumbar pain reduction than a conservative physical therapy or passive drug treatment. Especially, a lumbar extensor muscle strength therapy which stabilizes the lumbar vertebra is the most effective therapy to lessen the lumbar pain (3). In this respect, research on a lumbar extensor muscle strength therapy improving the muscular function of the muscle group around the lumbar vertebra, and relieving lumbar pain has been actively conducted (1, 3, 4). Meanwhile, a proprioceptive exercise is a balance exercise, which can be performed on a balance or wobble board. The proprioceptive exercise helps patients with ankle and back sprain strengthen their muscular function by more effectively maximizing proprioceptive stimulation compared to exercises performed on a flat surface (5). Furthermore, the proprioceptive exercise on an unbalanced surface stimulates the proprioceptive sense of our body and improves joint movement ability which controls muscle length, muscular tension, and joint position (6).

However, few studies have investigated the effectiveness of proprioceptive exercises with an unbalanced surface setting on pain reduction and joint movement enhancement in patients with lumbar pain. Thus, it is meaningful to conduct a study examining how a lumbar extension strength exercise on an unbalanced proprioceptive equipment relieves lumbar pain in a short period of time. More importantly, the current study possibly lays an academic foundation for future research that emphasizes a new proprioceptive stimulation such as surface conditions for an exercise program in this study to enhance the effectiveness of a lumbar extensor muscle strength therapy in patients with lumbar pain.

Fourteen female patients diagnosed with lumbar pain by orthopedists participated in this study. Informed consent to participate was obtained from all participants.

The participants underwent lumbar extensor muscle strength programs on either a flat surface or proprioceptive equipment (Jumper, TOGU, Germany). More specifically, the participants in both floor settings performed bare hand deadlifts (20 times x 5 sets) for a week. For the deadlifts, the participants were asked to stand with their feet hip-width apart, bend their upper body forward until their palms touch the floor, and slowly bring their upper body back to the standing position. For the clinical effectiveness of the exercise programs, Visual Analogue Scale (VAS) was utilized to analyze the degree of lumbar pain and Oswestry Disability Questionnaire (ODQ) was used to measure the functional disability of the participants.

All data were presented as mean ± standard deviation and analyzed using paired t-test. All analyses were performed using SPSS (ver. 23.0, Chicago, IL, USA). Statistical significance was set at *P*<0.05. The participant characteristics were as follows: flat surface group(female, n = 7; age, 51.16 ± 4.63 yr; height, 158.00 ± 8.49 cm; weight, 59.58 ± 8.49 kg) and proprioceptive group (female, n = 7; age, 50.25 ± 5.75 yr; height, 152.00 ± 4.26 cm; weight, 62.53 ± 6.96 kg). Results show that there were no significant differences between pre and post exercise intervention for VAS (*P*=0.363) and ODQ (*P*=0.518) in the flat surface group. However, there were significant differences between pre and post exercise intervention for VAS (*P*=0.001) and ODQ (*P*=0.023) in the proprioceptive group.

Compared to the exercise program on a flat surface, we found that lumbar extensor muscle strength exercise on a proprioceptive equipment reduced lumbar pain in a short period of time. Unlike traditional lumbar extensor muscle exercises, performed on a flat surface, the current study adopted a lumbar extensor muscle exercise utilizing a proprioceptive equipment, suggesting a new paradigm to treat patients with lumbar pain and contributing public health enhancement in terms of exercise program developments for lumbar pain patients.
